# THE IMPACT OF MUSIC AND MEMORYSM ON RESIDENT MOOD, BEHAVIORS, AND USE OF MEDICATIONS IN NURSING HOMES

**DOI:** 10.1093/geroni/igz038.2682

**Published:** 2019-11-08

**Authors:** Debra Bakerjian, Kristen Bettega, Ana Marin-Cachu, Leslie Azzis, Sandra Taylor

**Affiliations:** 1 Betty Irene Moore School of Nursing at UC Davis, Sacramento, California, United States; 2 University of California, Davis, Sacramento, California, United States

## Abstract

The numbers of Americans with dementia are projected to increase by 44.8% by 2025. Many of these patients are cared for in nursing homes (NH), 70% of NH residents with dementia have been reported to have significant behavioral or psychiatric symptoms (BPSD) that are often challenging to manage. Historically, the first lines of treatment for BPSD has been antipsychotic medications; however, serious adverse effects have been associated with these drugs. Our main aim was to study the effects of Music and MemorySM (M&M), a personalized music program, on improving behaviors and reducing antipsychotics and other medications in residents with dementia in participating NHs. This 3-year, quasi-experimental, mixed methods study used a cluster, randomized design in three phases. We used the Qualtrics Research Suite to create and disseminate a baseline survey and a 4-part quarterly survey thereafter. The quarterly survey collected select resident MDS data including diagnoses, medication use, pain, falls, mood and behaviors; how M&M was being implemented; resident use of M&M; organization level information. We also downloaded NH 5-Star quality rating quarterly. A total of 265 NH and 4,109 residents participated in the study. We found the odds of antipsychotic use declined by 11%, antianxiety medications by 17%, and antidepressants by 9% per quarter. The odds of residents exhibiting aggressive behaviors declined by 20% per quarter, depressive symptoms by 16% and residents reporting pain by 17%. Our findings indicate that M&M provides substantial benefit to NH residents, particularly in reduction of psychoactive medications and improving mood and behaviors.

